# Association between Serum Irisin Levels and Non-Alcoholic Fatty Liver Disease in Health Screen Examinees

**DOI:** 10.1371/journal.pone.0110680

**Published:** 2014-10-24

**Authors:** Eun Sung Choi, Mi Kyung Kim, Min Kyung Song, Jeong Min Kim, Eun Soo Kim, Woo Jin Chung, Kyung Sik Park, Kwang Bum Cho, Jae Seok Hwang, Byoung Kuk Jang

**Affiliations:** 1 Department of Internal Medicine, Keimyung University School of Medicine, Daegu, South Korea; 2 Department of Food Science and Nutrition, Graduate School, Keimyung University, Daegu, South Korea; 3 Institute for Cancer Research, Keimyung University, Daegu, South Korea; Northeast Ohio Medical University, United States of America

## Abstract

Irisin is a recently found myokine that aids obesity control and improves glucose homeostasis by acting on white adipose tissue cells and increases total energy consumption. The aim of this study was to evaluate serum irisin levels in patients with non-alcoholic fatty liver disease (NAFLD) and to compare these levels with those of normal controls. Among 595 health screen examinees who had visited our institute between January 2013 to March 2013, 355 patients (84 NAFLD patients and 271 normal controls) were enrolled depending on whether they gave written informed consents and their history of alcohol intake, blood tests, and abdominal ultrasonographic findings. Age; sex; laboratory test parameters; homeostasis model assessment-insulin resistance; and levels of leptin, adiponectin, and irisin were assessed. Serum irisin levels (ng/ml) were significantly higher in the NAFLD group than in normal controls (63.4±32.6 vs. 43.0±29.7, *p*<0.001) and higher in the mild fatty liver group than in the moderate-to-severe fatty liver group (68.3±38.2 vs. 56.6±21.2, *p*<0.001). Additionally, serum irisin levels were not different between the non-obese and obese groups (48.4±34.2 vs. 45.8±22.9, *p* = 0.492); however, the levels were significantly lowest in normal controls and highest in the mild fatty liver group in the non-obese (44.9±31.7 vs. 73.1±48.5 vs 59.7±18.0, *p*<0.001) and obese groups (35.0±17.0 vs. 62.9±21.2 vs. 54.6±23.3, *p*<0.001). Serum irisin levels were significantly higher in NAFLD patients, which is not consistent with the results of previously published studies. Therefore, more studies are needed to confirm the role of irisin in NAFLD.

## Introduction

Non-alcoholic fatty liver disease (NAFLD) involves a spectrum of hepatic diseases from hepatic steatosis to steatohepatitis and cirrhosis. While the percentage of NAFLD patients who progress to cirrhosis is low (5%), NAFLD is the most common cause of chronic liver disease because of the rapidly increasing prevalence of obesity [1]. The liver stores energy as glycogen and stores digested triglycerides in the form of fatty acids. Under stressful conditions such as obesity, excessive nutrition, and drug-induced liver injury, hepatocytes collect excess lipids; the prolonged storage of lipids leads to NAFLD [2].

NAFLD can be improved through weight reduction and exercise, without medical therapy [3]; however, the mechanism of improvement remains unknown even though many studies have been conducted to address this issue. Recently, irisin, a newly found myokine, has been recognized as an important factor in disease improvement. Irisin that is secreted in muscles during or after exercise expedites energy homeostasis and metabolism, improving obesity and glucose intolerance [3]–[5]. A laboratory study found that irisin induces white adipose cells to consume fat and product energy, and ultimately aids weight reduction and glucose intolerance [6]. Recent studies have shown that in patients with fatty liver disease, serum irisin levels are lower than that of healthy individuals [7].

Therefore, in this study, we examined serum irisin levels in NAFLD patients and healthy individuals and determined whether serum irisin levels are lower in NAFLD patients.

## Materials and Methods

Between January 2013 and March 2013, we performed a prospective analysis of 595 health screen examinees who visited the Keimyung University Dongsan Medical Center in Daegu, Korea. Viral hepatitis patients with positive serologic markers for hepatitis B or C, liver cirrhosis, malignant tumor, or other hepatobiliary diseases were excluded. One interviewer investigated the history of weekly alcohol intake, and 355 subjects who consumed <140 g of alcohol in case of women and <210 g of alcohol in case of men, each week, were enrolled. Among them, 84 examinees were diagnosed with NAFLD, and 271 examinees were designated as the normal controls. The diagnosis of NAFLD is based on a history of no or little weekly alcohol intake (<140 g for women and <210 g for men), the presence of hepatic steatosis by imaging, and the ruling out of other hepatic diseases [8]. We assessed several parameters such as age, sex, body measurements, and laboratory test parameters; abdominal ultrasonography was performed to verify the presence of steatosis and assess the degree of steatosis. We mainly used abdominal ultrasonography as the imaging tool, and a radiology expert carried out and registered the ultrasonographic results. Additionally, we analyzed serum irisin, leptin, and adiponectin levels with the ELISA kit (R&D, Minneapolis, MN, USA). We triple-checked these parameters and used mean values for analysis. We used the homeostasis model assessment-insulin resistance (HOMA-IR) value as the indicator of insulin resistance calculated using the following formula: fasting blood glucose (mg/dL) × insulin (mU/L)/(18×22.5) [9]. Furthermore, we examined liver function parameters and serum cholesterol levels, body mass index (BMI), abdominal circumference, thigh circumference, waist circumference, and hip circumference. We calculated BMI using the following formula: subject's weight (kg)/height (m)^2^. We applied the term “obese group” to subjects whose BMI was>25, and “non-obese group” to subjects whose BMI was <25, according to the World Health Organization.

Based on abdominal ultrasonography results, subjects were categorized as the control group, mild fatty liver group, and moderate-to-severe fatty liver group. We compared the demographic characteristics, body measurements, body composition, and laboratory study results between the groups. The grade of steatosis was assessed by a single radiologist. The normal liver parenchyma has echogenicity equal to or slightly greater than that of the renal cortex. In fatty liver disease, the liver shows higher echogenicity than the renal cortex because of fatty infiltration. In mild fatty liver disease, only the echogenicity increases; in moderate fatty liver disease, the walls of the portal vein branches are indistinct; and in severe fatty liver disease, the diaphragmatic outline is indistinct [8]. We applied the term “mild fatty liver group” to subjects whose ultrasonographic finding was consistent with mild fatty liver, and “moderate-to-severe fatty liver group” to subjects whose ultrasonographic findings were consistent with moderate and severe fatty liver. Metabolic syndrome was diagnosed when three or more of the following criteria were met: 1) abdominal circumference ≥90 cm for men and ≥85 cm for women, 2) triglyceride (TG) level ≥150 mg/dL, 3) decreased high-density lipoprotein (HDL) at <40 mg/dL for men and <50 mg/dL for women, 4) blood pressure ≥130/85 mmHg, and 5) fasting blood sugar (FBS) level ≥150 mg/dL, or history of diabetes mellitus (DM)[9].

### Ethics statement

The study protocol was approved by the institutional review board of Keimyung University Dongsan Hospital (IRB Number 12-221).

### Statistical analysis

Statistical analyses were performed with SPSS software version 18.0 for Windows (IBM, NY, USA). Continuous variables are expressed as mean ± SD. Categorical data were compared using the χ^2^ test. The Mann-Whitney U test and Kruskal-Wallis test were used to compare differences in continuous variables between each group. The correlation of irisin levels with other parameters was analyzed using Spearman correlation analysis. For all analyses, a *p* value ≤0.05 was considered to indicate statistical significance.

## Results

### Characteristics of study participants

Comparative data of the study groups are presented in Table 1. The mean age of the NAFLD group was significantly higher than that of the control group (*p* = 0.004), and the proportion of women was significantly higher in the control group (*p*<0.001). As expected, the mean BMI was significantly higher in the NAFLD group (*p*<0.001). Furthermore, there were statistically significant differences in FBS, insulin, HOMA, leptin, adiponectin, total cholesterol, TG, HDL, low-density lipoprotein (LDL), C-reactive protein (CRP), aspartate aminotransferase (AST), and alanine aminotransferase (ALT) levels between the two groups.

**Table 1:** Baseline characteristics of the subjects according to the study group.

	Control Group (n = 271)	NAFLD Group (n = 84)	*p* value
Age (yr)	44.4±9.9	48.0±9.8	0.004
Sex (Female)	215	46	<0.001
DM(n)	6	4	0.256
Metabolic syndrome(n)	17	20	<0.001
Irisin (ng/ml)	43.0±29.7	63.4±32.6	<0.001
BMI (kg/m^2^)	22.4±2.7	25.7±3.0	<0.001
FBS (mmol/L)	83.8±12.9	92.9±22.6	<0.001
Insulin (mIU/L)	3.5±2.4	6.4±4.1	<0.001
Homa	0.8±0.9	1.9±3.8	<0.001
Leptin (ng/ml)	2.2±1.5	3.3±3.0	0.002
Adiponectin (µg/ml)	6.2±3.9	3.6±2.5	<0.001
Systolic BP (mmHg)	115.8±17.7	124.5±14.9	<0.001
Diastolic BP (mmHg)	71.6±11.8	77.7±9.6	<0.001
Total cholesterol (mmol/L)	186.5±32.8	196.2±33.3	0.009
TG (mmol/L)	86.0±47.3	146.1±82.8	<0.001
HDL (mmol/L)	53.8±11.1	46.5±8.1	<0.001
LDL (mmol/L)	149.9±37.2	178.9±42.6	<0.001
CRP (mg/dL)	0.1±0.2	0.2±0.5	<0.001
AST (IU/L)	20.6±6.9	26.3±13.2	<0.001
ALT (IU/L)	17.4±8.1	33.4±24.9	<0.001
Waist (cm)	82.9±50.8	88.6±8.7	<0.001
Hip girth (cm)	90.6±7.6	96.0±6.9	<0.001
Weekly hours of exercise (min)	38.6±35.1	50.3±41.1	0.035

### Serum irisin levels according to the study group

**Figure 1:**
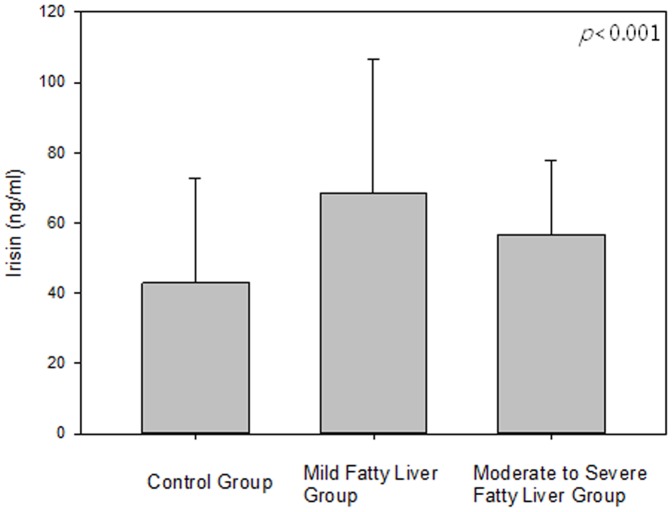
Serum irisin level in the control group and NAFLD group.

The serum irisin level in the NAFLD group was significantly higher than that of the control group (*p*<0.001) (Table 1). According to the severity of steatosis, the serum irisin level was highest in the mild fatty liver group and lowest in the control group (*p*<0.001) (Figure 1, Table 2). Furthermore, other parameters such as serum levels of FBS, insulin, HOMA, leptin, total cholesterol, TG, LDL, CRP, AST, and ALT; systolic blood pressure; and diastolic systolic blood pressure were significantly higher in the moderate-to-severe fatty liver group than the mild fatty liver group and normal controls (Table 2). The adiponectin and HDL levels were significantly lower in the NAFLD group than the control group, especially in the moderate-to-severe fatty liver group (*p*<0.001). The waist and hip circumferences were higher in the NAFLD group, especially in the severe fatty liver group (*p*<0.001) (Table 2).

**Table 2:** Baseline characteristics of the subject according to steatosis severity.

	Control Group (n = 271)	Mild Fatty Liver Group (n = 47)	Moderate to Severe Fatty Liver Group (n = 37)	*p* value
Age (yr)	44.4±9.9	47.1±9.1	49.2±10.6	0.012
Sex (Female)	215	29	17	<0.001
Irisin (ng/ml)	43.0±29.7	68.3±38.2	56.6±21.3	<0.001
BMI (kg/m^2^)	22.4±2.7	24.9±2.5	26.6±3.5	<0.001
FBS (mmol/L)	83.8±12.9	90.8±18.2	95.7±27.3	<0.001
Insulin (mIU/L)	3.5±2.4	5.9±3.7	6.9±4.4	<0.001
Homa	0.8±0.9	1.3±0.9	2.6±5.7	<0.001
Leptin (ng/ml)	2.2±1.5	3.3±3.3	3.3±2.6	0.009
Adiponectin (µg/ml)	6.2±3.9	3.7±2.6	3.4±2.5	<0.001
Systolic BP (mmHg)	115.8±17.7	122.2±16.2	127.3±12.7	<0.001
Diastolic BP (mmHg)	71.6±11.8	76.3±10.5	79.4±8.0	<0.001
Total cholesterol (mmol/L)	186.5±32.8	196.2±30.0	196.3±37.5	0.032
TG (mmol/L)	86.0±47.3	140.9±93.7	152.7±67.2	<0.001
HDL (mmol/L)	53.8±11.1	47.6±8.7	45.0±7.1	<0.001
LDL (mmol/L)	149.9±37.2	176.8±43.0	181.8±42.5	<0.001
CRP (mg/dL)	0.1±0.2	0.1±0.1	0.3±0.8	<0.001
AST (IU/L)	20.6±6.9	23.2±12.1	30.3±13.6	<0.001
ALT (IU/L)	17.4±8.1	26.4±22.5	42.3±25.4	<0.001
Waist (cm)	82.9±50.8	87.0±7.7	90.6±9.6	<0.001
Hip girth (cm)	90.6±7.6	95.0±6.3	97.3±7.5	<0.001
Weekly hours of exercise (min)	38.6±35.1	47.4±29.2	55.0±55.8	0.107

### Serum irisin levels according to the presence of obesity

**Figure 2:**
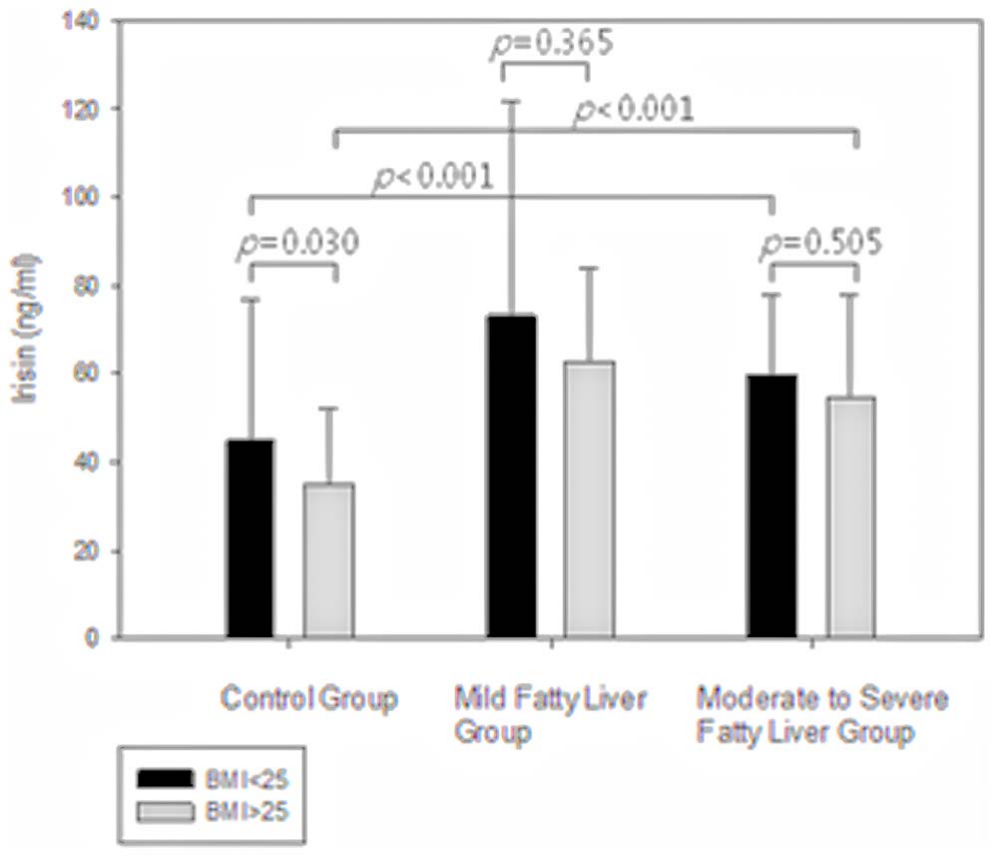
Serum irisin level of subjects according to obesity status.

Serum irisin levels (ng/ml) were not different between the non-obese and obese groups (48.4±34.2 vs. 45.8±22.9, *p* = 0.492) (Table 3). According to the severity of steatosis, the serum irisin level was lowest in normal controls and highest in the mild fatty liver group in the non-obese group (44.9±31.7 vs. 73.1±48.5 vs. 59.7±18.0, *p*<0.001). In the obese group (52 healthy controls and 46 NAFLD patients), the results were similar (35.0±17.0 vs. 62.9±21.2 vs. 54.6±23.3, *p*<0.001) (Figure 2, Table S1). According to the presence of obesity, in the control group, the serum irisin levels were significantly higher in the non-obese group than in the obese group (44.9±31.7 vs. 35.0±17.0, *p* = 0.030). On the other hand, in the NAFLD group including both the mild fatty liver group and the moderate-to-severe fatty liver group, the serum irisin level tended to be higher in the non-obese group than the obese group; however, the difference did not reach statistical significance (73.1±48.5 vs. 62.9±21.2, *p* = 0.365 and 59.7±18.0 vs. 54.6±23.3, *p* = 0.505) (Figure 2).

**Table 3:** Baseline characteristics of the subject according to the obesity status.

	Non-obese Group (n = 257)	Obese Group (n = 98)	*p* value
Age (yr)	43.9±9.7	48.6±10.0	<0.001
Sex (Female)	205	56	<0.001
DM (n)	345	10	0.472
Metabolic syndrome (n)	318	37	<0.001
NAFLD (n)	271	84	<0.001
Irisin (ng/ml)	48.4±34.2	45.8±22.9	0.492
FBS (mmol/L)	83.6±11.3	92.0±23.6	0.001
insulin (mIU/L)	3.4±1.9	6.1±4.5	<0.001
Homa	0.7±0.5	1.8±3.8	0.005
Leptin (ng/ml)	2.1±1.5	3.3±2.8	<0.001
Adiponectin (µg/ml)	5.9±3.8	4.4±3.2	<0.001
Systolic BP (mmHg)	114.7±15.5	126.2±19.6	<0.001
Diastolic BP (mmHg)	71.2±10.7	77.6±12.4	<0.001
Total cholesterol (mmol/L)	185.5±32.4	197.7±33.4	0.002
TG (mmol/L)	90.1±54.7	126.8±74.9	<0.001
HDL (mmol/L)	53.6±11.2	47.9±8.8	<0.001
LDL (mmol/L)	149.9±37.4	175.0±42.5	<0.001
CRP (mg/dL)	0.1±0.3	0.1±0.3	0.188
AST (IU/L)	20.8±7.9	25.1±11.3	0.001
ALT (IU/L)	18.5±12.5	28.2±20.1	<0.001
Waist (cm)	79.7±7.3	87.9±9.5	<0.001
Hip girth (cm)	90.4±7.7	95.8±6.8	<0.001
Weekly hours of exercise (min)	38.6±35.1	50.3±41.1	0.008

### Serum irisin levels according to the presence of type 2 DM

Among the study subjects, there were 10 type 2 DM patients. When DM patients were excluded from the control group, serum irisin levels were still significantly higher in the NAFLD group than in the control group (63.4±32.6 vs. 43.0±29.9, *p*<0.001).

### Serum irisin levels according to the presence of metabolic syndrome

There were 37 study subjects with metabolic syndrome: 17 (6.3%) in the control group and 20 (23.8%) in the NAFLD group. There was no difference in serum irisin levels between the subjects with and without metabolic syndrome (45.5±19.3 vs. 47.9±32.5, *p* = 0.663). Even after the metabolic syndrome patients were excluded from the control group, serum irisin levels were still significantly higher in the NAFLD group than the control group (63.4±32.6 vs. 43.7±30.3, *p*<0.001)

### Serum irisin levels according to the amount of exercise

The serum irisin level was not correlated with the quantity of exercise (r = −0.083, *p* = 0.254) (Table 4). Based on the weekly hours of exercise, we divided the subjects into three groups: the subjects in Group 1 (n = 195) did not exercise; those in Group 2 (n = 93) performed 1-h aerobic exercise weekly; and those in Group 3 (n = 67) performed>1-h aerobic exercise weekly. Serum irisin levels showed a slight decrease as the level of exercise increased (Group 1, 48.6±24.9; Group 2, 46.6±41.9; and Group 3, 46.5±32.4; *p* = 0.055). In Group 1, there were 42 NAFLD subjects (21.5%) and 44 obese subjects (22.6%). In Group 2, there were 20 NAFLD subjects (21.5%) and 28 obese subjects (30.1%). In Group 3, there were 22 NAFLD subjects (32.8%) and 26 obese subjects (38.8%).

**Table 4:** Correlations between serum irisin levels and other study parameters.

	r	r^2^	%	*p* value
BMI (kg/m^2^)	0.068	0.004624	0.4624	0.201
FBS (mmol/L)	0.054	0.002916	0.2916	0.313
Insulin (mIU/L)	0.204	0.041616	4.1616	<0.001
Homa	0.205	0.042025	4.2025	<0.001
Leptin (ng/ml)	−0.026	0.000676	0.0676	0.632
Adiponectin (µg/ml)	−0.086	0.007396	0.7396	0.109
Systolic BP (mmHg)	0.076	0.005776	0.5776	0.155
Diastolic BP (mmHg)	0.067	0.004489	0.4489	0.212
Total cholesterol (mmol/L)	−0.030	0.0009	0.09	0.580
TG (mmol/L)	0.086	0.007396	0.7396	0.107
HDL (mmol/L)	−0.055	0.003025	0.3025	0.307
LDL (mmol/L)	0.002	0.000004	0.0004	0.969
CRP (mg/dL)	0.065	0.004225	0.4225	0.221
AST (IU/L)	0.013	0.000169	0.0169	0.803
ALT (IU/L)	0.149	0.022201	2.2201	0.005
Waist (cm)	0.147	0.021609	2.1609	0.005
Hip girth (cm)	0.102	0.010404	1.0404	0.057
Weekly hours of exercise (min)	−0.083	0.006889	0.6889	0.254

### Serum irisin levels according to the sex

**Figure 3:**
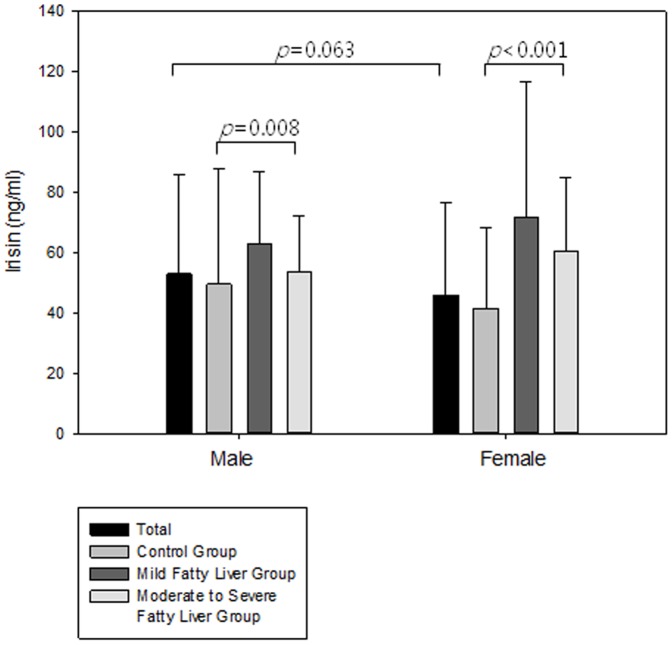
Serum irisin level according to sex.

Serum irisin levels tended to be higher in men than in women; however, there was no statistically significant difference between both sexes (52.9±32.7 vs. 45.8±30.9, *p* = 0.063). However, in both sex groups, there were significant differences between the sub-groups of NAFLD. In the male group, the serum irisin level was highest in the mild fatty liver group and lowest in the control group (49.5±38.2 vs. 62.9±23.6 vs. 53.5±18.7, *p* = 0.008). In the female group, the results were similar (41.3±26.9 vs. 71.6±45.1 vs. 60.5±24.3, *p*<0.001) (Figure 3, Table S2).

### Association between the serum irisin level and other parameters

The correlation analysis revealed that the serum level of irisin correlated with that of insulin (r = 0.204, *p*<0.001) and HOMA-IR (r = 0.205, *p*<0.001). However, when we analyzed the correlation in the control group, the mild fatty liver group and the moderate-to-severe fatty liver group separately, the serum level of irisin did not significantly correlate with that of insulin (r = 0.100, *p* = 0.100 vs. r = 0.186, *p* = 0.210 vs. r = −0.145, *p* = 0.413) and HOMA-IR (r = 0.092, *p* = 0.130 vs. r = 0.172, *p* = 0.247 vs. r = −0.079, *p* = 0.657). The serum irisin level did not correlate with other parameters such as leptin, adiponectin, triglyceride, AST, and ALT levels (Table 4).

## Discussion

Exercise is known to be beneficial in humans because it activates the transcriptional coactivator PGC1α in muscles and stimulates the expression of Fndc5, a membrane protein that is cleaved and secreted as irisin [4]. Irisin was firstly recognized as a muscle-derived glycosylated polypeptide in 2012 by Bostrom et al. [4]. Irisin acts on white adipose cells, which stimulate UCP1 expression and brown fat progress [4]. Huh et al. reported that the amount of muscle is the primary predictor of the serum level of irisin. Serum irisin levels increase in response to acute exercise and decrease after surgically induced weight loss and with decrease in body mass [3].

Polyzos et al. published a recent study that evaluated serum irisin levels in patients with NAFLD and controls; serum irisin levels were significantly lower in obese controls and NAFLD patients than in lean controls. Their results indicate that irisin may be independently and positively associated with the presence of portal inflammation [7].

However, in the present study, serum irisin levels in the obese group were increased, which was consistent with the results of a previous study, but were inconsistent with those of another study on NAFLD subjects. In our analysis, serum irisin levels did not decrease in NAFLD patients; instead, the serum irisin level in the NAFLD group was higher than that of the control group. Furthermore, the serum irisin level was higher in the mild NAFLD group than the severe NAFLD group. Zhang et al. reported that serum irisin levels were low in obese adults with NAFLD and reduced gradually with the increase in intrahepatic triglyceride levels [10]. However, they did not evaluate serum irisin levels in normal controls. In that respect, our results also reveal a gradual decrease in the serum irisin level according to the severity of steatosis. Therefore, we hypothesized that the serum irisin level increases as a defense mechanism in the early stage of NAFLD and then, decreases with the progression of NAFLD.

Although the mean BMI was higher in the NAFLD group, the serum irisin level was not different between the non-obese and obese groups. However, in the non-obese and obese groups, the serum irisin level was highest in the mild fatty liver group and lowest in the control group. Among the normal controls, the serum irisin level was lower in the obese group. In the mild fatty liver group and moderate-to-severe fatty liver group, there was no statistically significant difference in the serum irisin level between the obese and non-obese subjects; however, it tended to be lower in the obese group (Figure 2, Table S1). Consequently, we speculate that the increase of serum irisin in NAFLD patients is an independent process, not associated with BMI.

Liu et al. reported that serum irisin was significantly lower in type 2 DM subjects than in non-diabetic controls [11]. However, in the present study, the number of patients with type 2 DM was too small to analyze the relationship between serum irisin level and type 2 DM.

Park et al. reported that serum irisin levels were significantly higher in subjects with metabolic syndrome. They concluded that irisin is associated with metabolic syndrome independently from obesity. This association can be mediated not only by higher BMI or adiposity, but also by other mechanisms yet to be fully identified, including the direct effects on risk factors for metabolic syndrome [12]. However, although our data did not show a significant difference in serum irisin level between the metabolic syndrome group and normal control group, the number of subjects with metabolic syndrome was too low to reach a definite conclusion.

Our results showed that the quantity of exercise was not positively correlated with serum irisin levels. In fact, some authors recently reported that the regulation of irisin by exercise has been shown only in small sized cohorts, and the timing of irisin increase after exercise was uncertain [13], [14]. Kraemer et al. found temporarily elevated serum irisin levels in response to exercise, only during the first hour [15]. Hecksteden et al. failed to find an increase in serum irisin levels in healthy subjects after aerobic exercise [16]. In the present study, serum irisin levels showed a slight tendency to decrease with exercise; however, statistical significance was not reached. It is worth noting that there were more NAFLD patients and obese subjects in the exercise groups than in the non-exercise group. It is possible that the subjects who had NAFLD or were obese exercised more intensely than the healthy non-obese subjects before they were enrolled in the present study. Therefore, multiple complicated mechanisms may be involved in the relationship between serum irisin level and the quantity of exercise.

In the present study, serum irisin levels tended to be higher in men than in women. However, the number of women (n = 261) included in our study was much higher than the number of men (n = 94). Therefore, we analyzed the characteristics of subjects per sex. The proportion of NAFLD patients in the male group was 40.4% (38/94) and that of the female group was 17.6% (46/261). This factor may have influenced the higher irisin level in the male group. Nevertheless, there were significant differences between the subgroups of NAFLD in both sex groups (Figure 3, Table S2).

The serum irisin level is known to be lower in patients with fatty liver disease than in healthy individuals. However, recently published studies assert that serum irisin is not associated with BMI, age, and other biological parameters [14], [16] and that it is somewhat inversely associated. Our results would support these findings. One hypothesis from Silja et al. is that human Fndc5 is a transcribed pseudo-gene that has lost the ability to be effectively translated into full-length Fndc5 protein. Therefore, unlike in mice, the role of irisin is lost in humans [17].

Although our study included a relatively large number of subjects, it has several limitations. Data regarding the amount of alcohol intake and the weekly hours of exercise were obtained by personal history taking; therefore, data may have been underestimated or overestimated. We were unable to evaluate the intensity of exercise and the amount of muscle by imaging modality such as dual-energy X-ray absorptiometry or computed tomography. Finally, this study was limited by its cross-sectional design.

## Conclusion

In conclusion, although irisin is an interesting newly discovered protein, very little is known about it. It may be too premature to speculate an association between serum irisin levels and NAFLD. Our results showed that serum irisin levels were higher in NAFLD patients, which is not consistent with the results of previously published studies. Future studies will be required to ascertain the precise mechanisms regulating the effects of irisin on energy expenditure.

## Supporting Information

Table S1 **Serum irisin level of subjects according to obesity status.**
(DOCX)

Table S2 **Serum irisin level according to sex.**
(DOCX)
