# Supplementary material for: Association between Serum Irisin Levels and Non-Alcoholic Fatty Liver Disease in Health Screen Examinees
Source: PLoS One. 2014 Oct 24;9(10):e110680. doi: 10.1371/journal.pone.0110680 (PMC4208808; doi:10.1371/journal.pone.0110680)
Supplement: Table S1 — Serum irisin level of subjects according to obesity status. (DOCX) [file pone.0110680.s001.docx]

Supplementary Table 1. Serum irisin level of subjects according to obesity status

|  | Non-obese Group (n=257) | | | | Obese Group (n=98) | | | |
| --- | --- | --- | --- | --- | --- | --- | --- | --- |
|  | Control Group (n=219) | Mild Fatty Liver Group (n=25) | Moderate to Severe Fatty Liver Group (n=13) | *p* value | Control Group (n=52) | Mild Fatty Liver Group (n=22) | Moderate to Severe Fatty Liver Group (n=24) | *p* value |
| Age (yr) | 43.3±9.5 | 46.1±8.3 | 50.9±12.3 | 0.048 | 48.9±10.4 | 48.2±9.9 | 48.3±9.8 | 0.977 |
| Sex (Female) | 182 | 18 | 5 | <0.001 | 33 | 11 | 12 | 0.405 |
| DM (n) | 4 | 2 | 0 | 0.130 | 2 | 1 | 1 | 0.990 |
| Metabolic syndrome (n) | 9 | 3 | 1 | 0.212 | 8 | 4 | 12 | 0.004 |
| Irisin (ng/ml) | 44.9±31.7 | 73.1±48.5 | 59.7±18.0 | <0.001 | 35.0±17.0 | 62.9±21.2 | 54.6±23.3 | <0.001 |
| FBS (mmol/L) | 82.3±7.9 | 91.1±23.8 | 91.2±14.5 | 0.029 | 89.9±23.6 | 90.4±8.7 | 98.1±32.2 | 0.070 |
| insulin (mIU/L) | 3.2±1.6 | 5.1±3.3 | 4.2±2.3 | 0.004 | 4.7±4.2 | 6.9±4.1 | 8.4±4.6 | <0.001 |
| Homa-IR | 0.7±0.4 | 1.2±0.8 | 0.9±0.7 | 0.002 | 1.2±1.9 | 1.5±0.9 | 3.5±6.9 | <0.001 |
| Leptin (ng/ml) | 2.0±1.5 | 2.6±1.7 | 2.4±1.9 | 0.164 | 2.7±1.7 | 4.0±4.4 | 3.7±2.9 | 0.486 |
| Adiponectin (µg/ml) | 6.3±3.9 | 4.6±3.0 | 3.9±3.4 | 0.002 | 5.7±3.7 | 2.6±1.3 | 3.1±1.8 | <0.001 |
| Systolic BP (mmHg) | 113.8±15.9 | 117.8±12.6 | 123.5±10.9 | 0.014 | 124.2±22.3 | 127.2±18.6 | 129.4±13.4 | 0.511 |
| Diastolic BP (mmHg) | 70.5±10.9 | 74.5±8.9 | 76.8±6.5 | 0.007 | 75.9±13.8 | 78.3±12.0 | 80.9±8.5 | 0.204 |
| Total cholesterol (mmol/L) | 185.1±32.9 | 190.8±27.4 | 181.8±34.6 | 0.509 | 192.7±31.6 | 202.3±32.3 | 204.1±37.4 | 0.297 |
| TG (mmol/L) | 81.1±40.8 | 150.5±95.7 | 126.0±68.9 | <0.001 | 106.7±64.8 | 130.1±92.3 | 167.1±63.0 | <0.001 |
| HDL (mmol/L) | 54.9±11.2 | 46.7±8.7 | 45.5±7.3 | <0.001 | 49.2±9.3 | 48.5±8.7 | 44.7±7.2 | 0.116 |
| LDL (mmol/L) | 146.4±35.6 | 174.2±42.0 | 161.5±40.9 | 0.002 | 164.8±40.3 | 179.7±44.8 | 192.8±39.9 | 0.019 |
| CRP (mg/dL) | 0.04±0.1 | 0.08±0.1 | 0.6±1.2 | <0.001 | 0.1±0.4 | 0.1±0.1 | 0.2±0.3 | 0.022 |
| AST (IU/L) | 20.2±6.4 | 22.7±14.4 | 26.2±11.9 | 0.008 | 22.2±8.9 | 23.7±9.2 | 32.5±14.2 | 0.001 |
| ALT (IU/L) | 16.8±8.2 | 25.9±27.9 | 32.5±14.3 | <0.001 | 19.8±7.6 | 26.9±14.8 | 47.6±28.6 | <0.001 |
| Waist (cm) | 79.1±7.4 | 83.3±6.6 | 82.2±5.2 | 0.005 | 83.3±8.3 | 91.2±6.7 | 95.2±8.3 | <0.001 |
| Hip girth (cm) | 90.1±7.9 | 93.2±6.8 | 90.6±3.8 | 0.081 | 92.9±6.1 | 97.1±5.0 | 100.9±6.4 | <0.001 |
| Weekly hours of exercise (min) | 36.7±30.1 | 46.6±22.6 | 23.3±22.5 | 0.107 | 43.6±46.3 | 48.5±36.7 | 70.8±61.4 | 0.136 |
