# Supplementary material for: Association between Serum Irisin Levels and Non-Alcoholic Fatty Liver Disease in Health Screen Examinees
Source: PLoS One. 2014 Oct 24;9(10):e110680. doi: 10.1371/journal.pone.0110680 (PMC4208808; doi:10.1371/journal.pone.0110680)
Supplement: Table S2 — Serum irisin level according to sex. (DOCX) [file pone.0110680.s002.docx]

Supplementary Table 2. Serum irisin level according to sex

|  | Male (n=94) | | | | Female (n=261) | | | |
| --- | --- | --- | --- | --- | --- | --- | --- | --- |
|  | Control Group (n=56) | Mild Fatty Liver Group (n=18) | Moderate to Severe Fatty Liver Group (n=20) | *p* value | Control Group (n=215) | Mild Fatty Liver Group (n=29) | Moderate to Severe Fatty Liver Group (n=17) | *p* value |
| Age (yr) | 47.4±10.9 | 48.7±10.4 | 48.4±11.3 | 0.843 | 43.6±9.5 | 46.1±8.2 | 50.2±9.9 | 0.015 |
| DM (n) | 1 | 1 | 0 | 0.477 | 5 | 2 | 1 | 0.319 |
| Metabolic syndrome (n) | 4 | 2 | 7 | 0.008 | 13 | 5 | 6 | <0.001 |
| BMI (kg/m^2^) | 23.7±2.6 | 25.6±1.7 | 26.4±3.6 | 0.001 | 22.1±2.6 | 24.6±2.9 | 26.7±3.3 | <0.001 |
| Irisin (ng/ml) | 49.5±38.2 | 62.9±23.6 | 53.5±18.7 | 0.008 | 41.3±26.9 | 71.6±45.1 | 60.5±24.3 | <0.001 |
| FBS (mmol/L) | 84.0±8.5 | 93.4±15.9 | 97.6±33.3 | 0.015 | 83.7±13.8 | 89.1±19.5 | 93.4±18.7 | 0.004 |
| insulin (mIU/L) | 3.4±1.7 | 5.7±4.4 | 6.6±5.1 | 0.008 | 3.5±2.6 | 6.1±3.4 | 7.3±3.6 | <0.001 |
| Homa-IR | 0.7±0.4 | 1.3±1.0 | 1.9±2.6 | 0.003 | 0.8±1.0 | 1.4±0.8 | 3.5±7.9 | <0.001 |
| Leptin (ng/ml) | 1.2±0.8 | 1.9±1.7 | 1.8±1.4 | 0.079 | 2.4±1.6 | 4.2±3.8 | 4.9±2.8 | <0.001 |
| Adiponectin (µg/ml) | 4.6±2.6 | 3.9±3.7 | 3.0±2.6 | 0.008 | 6.6±4.0 | 3.6±1.6 | 3.8±2.2 | <0.001 |
| Systolic BP (mmHg) | 120.3±27.7 | 127.4±19.2 | 127.5±11.8 | 0.439 | 114.6±13.9 | 119.0±13.4 | 127.1±14.2 | 0.001 |
| Diastolic BP (mmHg) | 74.7±17.6 | 80.5±11.2 | 79.5±6.0 | 0.356 | 70.7±9.5 | 73.7±9.3 | 79.4±10.1 | 0.003 |
| Total cholesterol (mmol/L) | 193.8±31.9 | 203.2±34.2 | 183.0±40.9 | 0.162 | 184.6±32.8 | 191.8±26.8 | 211.9±26.4 | 0.001 |
| TG (mmol/L) | 113.3±71.9 | 170.8±131.8 | 157.0±72.3 | 0.009 | 78.9±35.4 | 122.4±54.3 | 147.5±62.6 | <0.001 |
| HDL (mmol/L) | 48.6±10.4 | 45.4±7.6 | 41.6±6.4 | 0.015 | 55.1±10.8 | 48.9±9.1 | 49.1±5.8 | 0.001 |
| LDL (mmol/L) | 167.9±40.6 | 191.9±50.8 | 172.8±47.7 | 0.237 | 145.3±34.9 | 167.4±35.1 | 192.4±33.9 | <0.001 |
| CRP (mg/dL) | 0.1±0.2 | 0.1±0.1 | 0.3±0.9 | 0.119 | 0.1±0.2 | 0.1±0.1 | 0.3±0.6 | <0.001 |
| AST (IU/L) | 24.4±9.6 | 27.7±16.0 | 28.7±9.9 | 0.033 | 19.6±5.8 | 20.3±8.0 | 32.1±17.2 | 0.001 |
| ALT (IU/L) | 23.8±10.4 | 37.3±31.6 | 45.9±28.1 | <0.001 | 15.7±6.5 | 19.6±10.1 | 38.0±21.8 | <0.001 |
| Waist (cm) | 82.1±8.0 | 89.3±3.9 | 90.6±9.6 | <0.001 | 79.4±7.6 | 85.6±9.0 | 90.6±9.9 | <0.001 |
| Hip girth (cm) | 91.8±6.1 | 96.8±3.1 | 97.9±7.4 | <0.001 | 90.3±7.9 | 93.9±7.4 | 96.6±7.8 | 0.001 |
| Weekly hours of exercise (min) | 47.2±35.8 | 48.2±21.8 | 66.0±71.9 | 0.859 | 35.6±34.5 | 46.9±33.5 | 41.3±22.9 | 0.203 |
